# Investigation of clinicopathological parameters and expression of COX-2, bcl-2, PCNA, and p53 in primary and recurrent sporadic odontogenic keratocysts

**DOI:** 10.1007/s00784-018-2400-7

**Published:** 2018-03-05

**Authors:** Tomasz Kaczmarzyk, Konrad Kisielowski, Rafał Koszowski, Magdalena Rynkiewicz, Ewa Gawełek, Karolina Babiuch, Anna Bednarczyk, Bogna Drozdzowska

**Affiliations:** 1 Department of Oral Surgery, Institute of Dentistry, Medical College, Jagiellonian University, ul. Montelupich 4, 31-155 Kraków, Poland; 2 Chair of Periodontology and Clinical Pathology of the Oral Cavity, Institute of Dentistry, Medical College, Jagiellonian University, ul. Montelupich 4, 31-155 Kraków, Poland; 3 Academic Center of Dentistry and Specialized Medicine, Plac Akademicki 17, 41-902 Bytom, Poland; 4 Department of Pathomorphology, School of Medicine with the Division of Dentistry in Zabrze, Medical University of Silesia, ul. 3 Maja 13/15, 41-800 Zabrze, Poland

**Keywords:** Odontogenic keratocyst, Keratocystic odontogenic tumor, COX-2, bcl-2, PCNA, p53

## Abstract

**Objectives:**

Odontogenic keratocyst (OKC) presents considerable variation in aggressiveness and propensity for recurrence, yet hitherto, no explicit clinicopathological features have been determined to clearly demonstrate the potential for relapse. This retrospective study aims to investigate the prognostic relevance of various clinicopathological features as well as immunoexpression of COX-2, bcl-2, PCNA, and p53 in sporadic OKC.

**Materials and methods:**

Among 41 patients with OKC treated by enucleation, the frequency of recurrence for various clinicopathological features as well as immunoexpression for COX-2, bcl-2, PCNA, and p53 was evaluated.

**Results:**

The mean follow-up was 8.49 years, and recurrences were ascertained in 29.27% of cases. We found significant differences between recurrent and non-recurrent cysts in terms of multilocularity (*P* = 0.029), cortical perforation (*P* = 0.001), and lesion size (*P* < 0.001). Hazard risk for the recurrence was 3.362 (95% CI 1.066–10.598) for multilocular cysts, 7.801 (95% CI 2.1–28.985) for evidence of cortical perforation, and 1.004 (1.002–1.006) for 1 mm^2^ of lesion size on panoramic radiographs. We also found that immunoexpression of PCNA significantly correlates with the radiographic evidence of cortical perforation (*P* = 0.048) and that there is significant positive correlation between expression of COX-2 and bcl-2 (*P* = 0.001) as well as significant negative correlation between immunoexpression of COX-2 and age (*P* = 0.002). None of the other analyzed factors were associated with the recurrence.

**Conclusions:**

Larger size, multilocularity, and cortical perforation in sporadic OKC may be correlated with the relapse.

**Clinical relevance:**

Immunohistochemical analyses of COX-2, bcl-2, PCNA, and p53 lack prognostic utility in sporadic OKC.

## Introduction

The keratocystic odontogenic tumor (KCOT) was defined by the World Health Organization (WHO) in 2005 as a benign, intraosseous tumor of odontogenic origin, with characteristic lining of parakeratinized stratified squamous epithelium and a potential for aggressive, infiltrative behavior [1]. It was believed that this term better reflected the neoplastic nature of this lesion in comparison with the previous term of odontogenic keratocyst (OKC). However, only recently, WHO decided to move KCOT back into the cyst category as OKC [2]. Mutations in the PTCH gene which were the main evidence for 2005 classification of the lesion into a group of odontogenic tumors are currently believed not to be KCOT-specific, and there are documented cases of spontaneous regression of the lesion following only its partial removal (decompression or marsupialization) which contradicts the main concept of autonomy of neoplasm continuing to grow after the stimulus which produced it is removed [3].

OKC is a quite common clinical finding as it accounts for 10–20% of odontogenic cysts and is the third most common cyst of the jaws [2]. A number of treatment modalities have been recommended for OKC, including simple enucleation, enucleation followed by Carnoy’s solution application, and decompression as being the most commonly employed [4]. Several systematic reviews and meta-analyses have been produced to establish which method results in the lowest recurrence rate; however, their conclusions are ambiguous and the golden standard treatment for OKC remains hotly debated [5–7]. Among reasons for this, the considerable variation in aggressiveness and propensity for recurrence of OKC is widely acknowledged [6, 8], and hitherto, no explicit clinical and radiological features have been determined to clearly demonstrate the potential for the aggressive growth and relapse of the lesion. Hence, the researchers have tried to elucidate the relationship between the expression of various molecular markers and the biological potential of OKC. Some studies have shown that the local aggressiveness of OKC and its propensity for recurrence may be consistent with the expression of several markers of epithelial cell proliferation and apoptosis, including Ki-67 [8], bcl-2 [9], cyclin D1 [9], p53 [9], and PCNA [9, 10].

Studies have demonstrated that cyclooxygenase-2 (COX-2) levels are elevated in various tumors, particularly those involving the gastrointestinal tract, lung, skin, urinary bladder, and prostate as well as head and neck [11]. The regulation of COX-2 expression is crucial for prostaglandin E_2_ (PGE_2_) synthesis, and its upregulation may increase the synthesis of prostaglandins (PGs), thus promoting cell proliferation and angiogenesis, and inhibiting immunosurveillance [12]. Recent studies revealed that COX-2 is also overexpressed in OKC, thus suggesting that it may be an important marker involved in the biologic behavior of the OKC [12, 13]. It has also been found that COX-2 expression in OKC is significantly decreased following decompression [13] and that its expression is not correlated with clinical features of the lesion [12]. However, prognostic significance of immunopositivity of COX-2 in OKC has not hitherto been examined.

Overexpression of the COX-2 gene inhibits apoptosis and enhances invasiveness which has been attributed, to some extent, to increased level of antiapoptotic protein B cell lymphoma 2 (bcl-2) [11] which, in contrast to radicular and dentigerous cysts, is upregulated in OKC [14]. Apart from apoptosis-related factors involved in development of OKC, proliferative activity of its lining epithelium has been the subject of various investigations. Such studies concluded that proliferative activity of OKC epithelium is significantly higher than in other types of odontogenic cysts, as shown by immunopositivity of several markers, including proliferating cell nuclear antigen (PCNA) [10] and p53 [15]. p53 status is associated with COX-2 and PCNA expression [15] as well as some clinical features of OKC, such as recurrence and association with nevoid basal cell carcinoma syndrome (NBCCS) [16].

Based on the aforementioned findings, the primary objective of this study was therefore to identify the association between recurrence-free survival and protein expression of COX-2, bcl-2, PCNA, and p53 by immunohistochemistry so as to find a prognostic marker in OKC. The secondary objective was to elucidate any relationship between clinicopathological findings and recurrence as well as immunoexpression of COX-2, bcl-2, PCNA, and p53.

## Materials and methods

Sixty-five cases diagnosed with OKC or KCOT by histopathology and confirming to 2017 WHO criteria of OKC were selected for this retrospective study. Patients were treated between 1997 and 2015 in two units of oral surgery in Poland (Department of Oral Surgery at the Medical College of the Jagiellonian University in Krakow and Academic Center of Dentistry and Specialized Medicine in Bytom). All cases were treated by simple enucleation. This method refers to surgically shelling the cyst out of the bone to remove the entire lesion without leaving any macroscopic remnants [6, 17]. Following enucleation, no bone regeneration graft materials were applied. Cases treated by means of enucleation with any adjunct procedure (e.g., peripheral ostectomy, Carnoy’s solution, liquid nitrogen) or method using partial removal of the cyst lining (marsupialization or decompression) were not included in this analysis. The surgical technique used was standardized among surgeons performing enucleation. Among 65 subjects, 12 cases were lost to follow-up after 6 months of surgical treatment and were thus excluded. Twelve subjects with NBCCS-associated OKCs were also excluded from the analysis. A total of 41 subjects treated for OKC were finally reviewed. Ethical approvals were obtained from Bioethical Committee of the Jagiellonian University (KBET/58/B/2012) and Bioethical Committee of the Medical University of Silesia (KNW/0022/KB/275/16).

Panoramic and occlusal radiographs were taken at the time of diagnosis and at follow-up visits (quarterly for the first post-operative year and semiannually from the subsequent year). In most cases, CT or CBCT scans were also performed. During follow-up period, gradual bone regeneration has been radiologically observed. At follow-up appointments, all patients were evaluated for any symptoms of recurrence (e.g., clinical bone expansion and/or radiological detection of suspicious radiolucent zone in the area of previously enucleated cyst). The relapse period was defined from the time of surgery to the time of detection of recurrence. Each recurrent lesion was histopathologically confirmed to meet the WHO microscopic criteria of OKC [2].

The following clinicoradiological data were retrieved: age, gender, observation period and evidence of relapse, cyst location, size of entire cystic area in radiographs (calculated by multiplying the major and minor axes on panoramic radiographs), tumor variant (uni- or multilocular), and radiological evidence of cortical perforation (destruction of cortex by a lytic process) which has been described as a possible imaging feature of OKC [1]. Histopathological characteristics analyzed were as follows: existence of daughter cysts, presence and intensity of inflammation, and epithelial dysplasia. Cyst location was divided into the following anatomical sites: anterior maxilla, posterior maxilla, anterior mandible, and posterior mandible. The boundary line between anterior and posterior maxilla/mandible was the posterior surface of the second premolar. Observation periods and any evidence of relapse were also retrieved.

Formalin-fixed, parafin-embedded archival blocks were sectioned and stained by hematoxylin-eosin (H&E). Slides with 5-μm-thick tissue sections were used to confirm a diagnosis of OKC and to evaluate histopathological characteristics (existence of daughter cysts, presence and intensity of inflammation, and epithelial dysplasia) using a light microscope. The intensity of inflammation was measured by counting inflammatory cells and stratified by using 4-grade scoring system according to Kuroyanagi et al. [8]: grade 0—no inflammation, grade 1—less than 15 cells, grade 2—15–50 cells, and grade 3—over 50 cells in 10 high-power fields (HPFs).

For immunohistochemical analysis, paraffin-embedded 3-μm-thick tissue sections were used. After antigen retrieval, sections were blocked by incubation with 3% H_2_O_2_ and the next slides were incubated with following antibodies:

- Rabbit monoclonal COX-2 (SP21, Cell Marque, Rocklin, CA, USA; 1:200) room temp., 45 min;
- Rabbit monoclonal bcl-2 (E17, Cell Marque, Rocklin, CA, USA; 1:300) 4 °C, overnight;
- Mouse monoclonal p53 (DO7, Cell Marque, Rocklin, CA, USA; 1:100) room temp., 50 min; and
- Rabbit polyclonal PCNA (RB-9055-P0, Thermo Scientific, Fremont, CA, USA; 1:700) room temp., 30 min.

Subsequently, sections were treated with Primary Antibody Amplifier Quanto followed by HRP Polymer Quanto (Thermo Scientific, Fremont, CA, USA). The slides were then stained with 3-3′-diaminobenzidine DAB Quanto (Thermo Scientific, Fremont, CA, USA). Eventually, tissue sections were counterstained with hematoxylin, dehydrated, and covered with coverslips for further analysis. Tonsil was used as positive control for bcl-2, p53, and PCNA. For negative control, sections were treated as above but without the primary antibody. Cellular staining patterns in the epithelium of OKC were cytoplasmic for COX-2, membranous-cytoplasmic for bcl-2, or nuclear for p53 and PCNA.

Semiquantitative assessment of COX-2 and bcl-2 using 4-grade scoring system was used: grade 0—negative reaction (no staining cells), grade 1—staining of 1–25% cells, grade 2—staining of 26–50% cells, and grade 3—more than 50% of staining cells in the epithelium. For PCNA and p53, quantitative assessment was applied. The number of positive staining cells was evaluated per 1000 epithelial cells and expressed in percentage. All histopathological and immunohistochemical evaluations were made by board-certified specialist in pathomorphology (B.D.).

All clinicopathological and immunohistochemical features were evaluated against the frequency of recurrence. Results were reported as mean (± SD) or median (range) where appropriate. Differences in clinicoradiological and pathological data were analyzed using Fisher’s exact test, Student’s *t* test, and Mann-Whitney test where appropriate. Cox proportional hazard model for time-dependent variables was implemented to evaluate hazard ratio and 95% confidence intervals (CI) as estimates of hazard risk (HR) for a recurrence potential. Associations between clinicoradiological and histopathological features and immunoexpression of COX-2, bcl-2, PCNA, and p53 were determined by Spearman correlation analysis. *P* values less than 0.05 were considered significant. All analyses were performed using the R Project for Statistical Computing.

## Results

The mean age of patients at the time of surgery was 40.24 (± 18.3) years, and the mean follow-up was 8.49 (± 4.34) years. Recurrences were ascertained in 12 (29.27%) cases. The mean recurrence period was 3.92 (± 2.61) years. The mean age of patients with non-recurrent cysts at the time of surgery was 40.76 (± 17.2) years, and of those with recurrent cysts was 39 (± 21.52) years (*P* = 0.784; Student’s *t* test). The age of a patient at the time of diagnosis was not associated with recurrence [HR 0.997, 95% CI 0.966–1.03 (*P* = 0.855)].

Clinicoradiological and histopathological characteristics of the cases are presented in Table 1. The median size of recurrent cysts was 782.5 (range 673.5–978.5) mm^2^, and of non-recurrent cysts was 375 (330–420) mm^2^ (*P* < 0.001; Mann-Whitney test). The size of lesion was significantly associated with cyst relapse [HR 1.004, 95% CI 1.002–1.006 (*P* < 0.001)] by the Cox proportional hazard model. We also found that radiological evidence of multilocularity as well as radiological evidence of cortical perforation was significantly associated with recurrence of OKC (Table 1). None of the other clinicoradiological findings were significantly associated with relapse of OKC (Table 1).

**Table 1:** Clinicoradiological and histopathological characteristics of the cases with hazard risks for recurrence of OKC

	Recurrent cysts (*n* = 12)	Non-recurrent cysts (*n* = 29)	Total (*n* = 41)	*P* value (Fisher’s exact test)	Cox proportional hazard model	
					HR (95% CI)	*P* value
Gender						
Male	9 (75.0%)	12 (41.4%)	21 (51.2%)	0.085	1.00	
Female	3 (25.0%)	17 (58.6%)	20 (48.8%)		0.311 (0.084–1.15)	0.08
Cyst location						
Posterior mandible	5 (41.7%)	15 (51.7%)	20 (48.8%)	0.394	1.00	
Anterior mandible	4 (33.3%)	5 (17.2%)	9 (22.0%)		2.162 (0.578–8.081)	0.252
Anterior maxilla	3 (25.0%)	5 (17.2%)	8 (19.5%)		1.645 (0.392–6.898)	0.496
Posterior maxilla	0 (0.0%)	4 (13.8%)	4 (9.75%)		NR	
Cyst variant						
Unilocular	5 (41.7%)	23 (79.3%)	28 (68.3%)	0.029*	1.00	
Multilocular	7 (58.3%)	6 (20.7%)	13 (31.7%)		3.362 (1.066–10.598)	0.039*
Cortical perforation						
Negative	3 (25.0%)	24 (82.8%)	27 (65.8%)	0.001*	1.00	
Positive	9 (75.0%)	5 (17.2%)	14 (34.2%)		7.801 (2.1–28.985)	0.002*
Inflammatory score						
Grade 0	6 (50.0%)	12 (41.4%)	18 (44.0%)	0.821	1.00	
Grade 1	3 (25.0%)	11 (37.9%)	14 (34.2%)		0.572 (0.143–2.291)	0.43
Grade 2	2 (16.7%)	5 (17.2%)	7 (17.1%)		0.831 (0.168–4.122)	0.821
Grade 3	1 (8.3%)	1 (3.45%)	2 (4.9%)		2.062 (0.245–17.345)	0.505
Daughter cysts						
Negative	9 (75.0%)	22 (75.9%)	31 (75.6%)	0.864	1.00	
Positive	3 (25.0%)	7 (24.1%)	10 (24.4%)		1.26 (0.34–4.667)	0.729
Epithelial dysplasia						
Negative	11 (91.7%)	28 (96.6%)	39 (95.1%)	0.505	1.00	
Positive	1 (8.3%)	1 (3.4%)	2 (4.9%)		1.518 (0.196–11.777)	0.69

Asterisks denote statistical significance

*NR*, no recurrence; *HR*, hazard ratio; *CI*, confidence interval

According to the WHO definition of OKC [2], every case analyzed in this study was lined with parakeratinized stratified squamous epithelium. Epithelial dysplasia was a very rare finding. Only low-grade squamous epithelial dysplasia was diagnosed in one non-recurrent and in one recurrent cysts. Inflammation was more common in recurrent (50%) than in non-recurrent (41.4%) lesions. Additionally, among the recurrent cases, the strong intensity of inflammation (grade 3) was observed twice as often as in non-recurrent lesions. In turn, the presence of daughter cysts was comparable between recurrent and non-recurrent OKCs (25 and 24.1%, respectively). None of the analyzed histopathological findings were significantly associated with recurrence (Table 1).

**Fig. 1:**
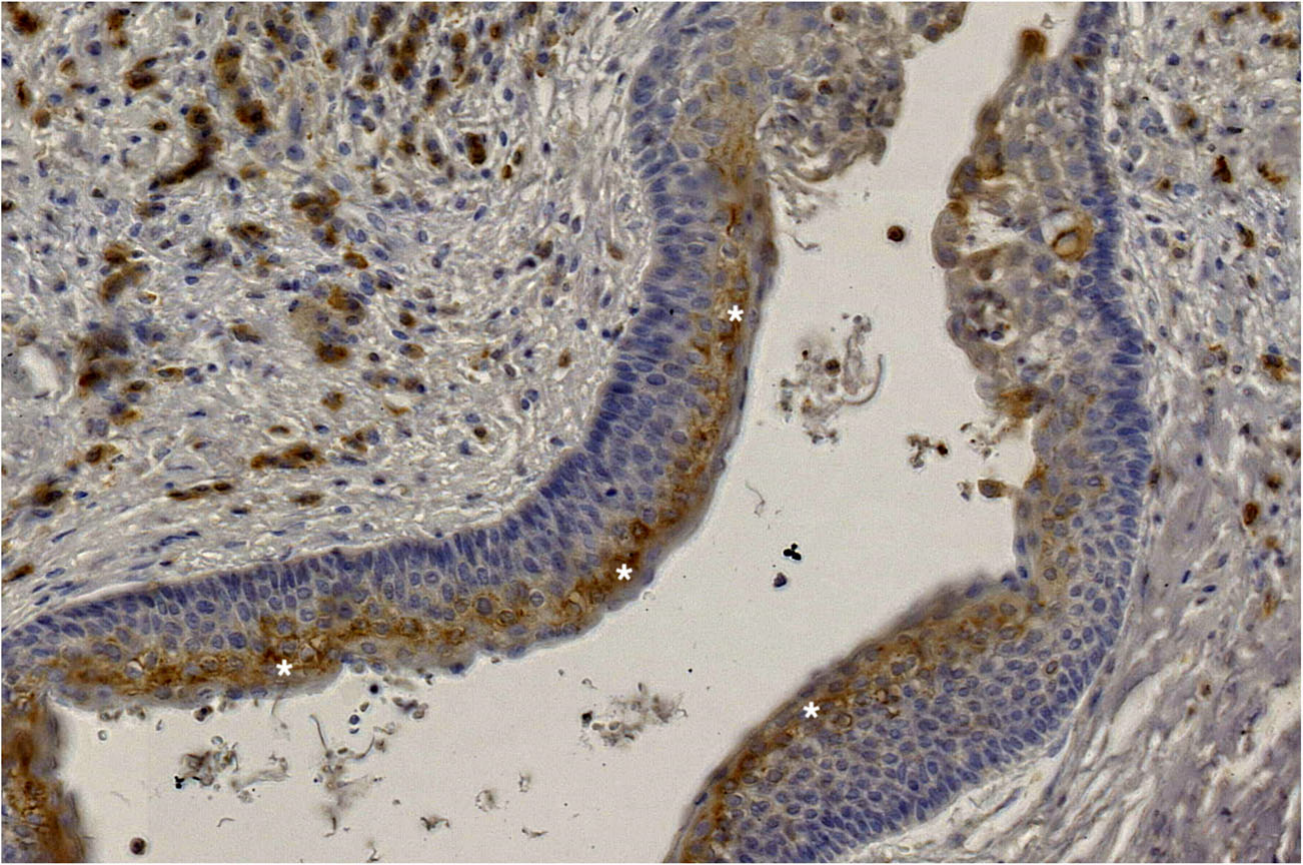
COX-2—cytoplasmic brown staining in the superficial layer (asterisks) of the epithelial lining of OKC [×60]

**Fig. 2:**
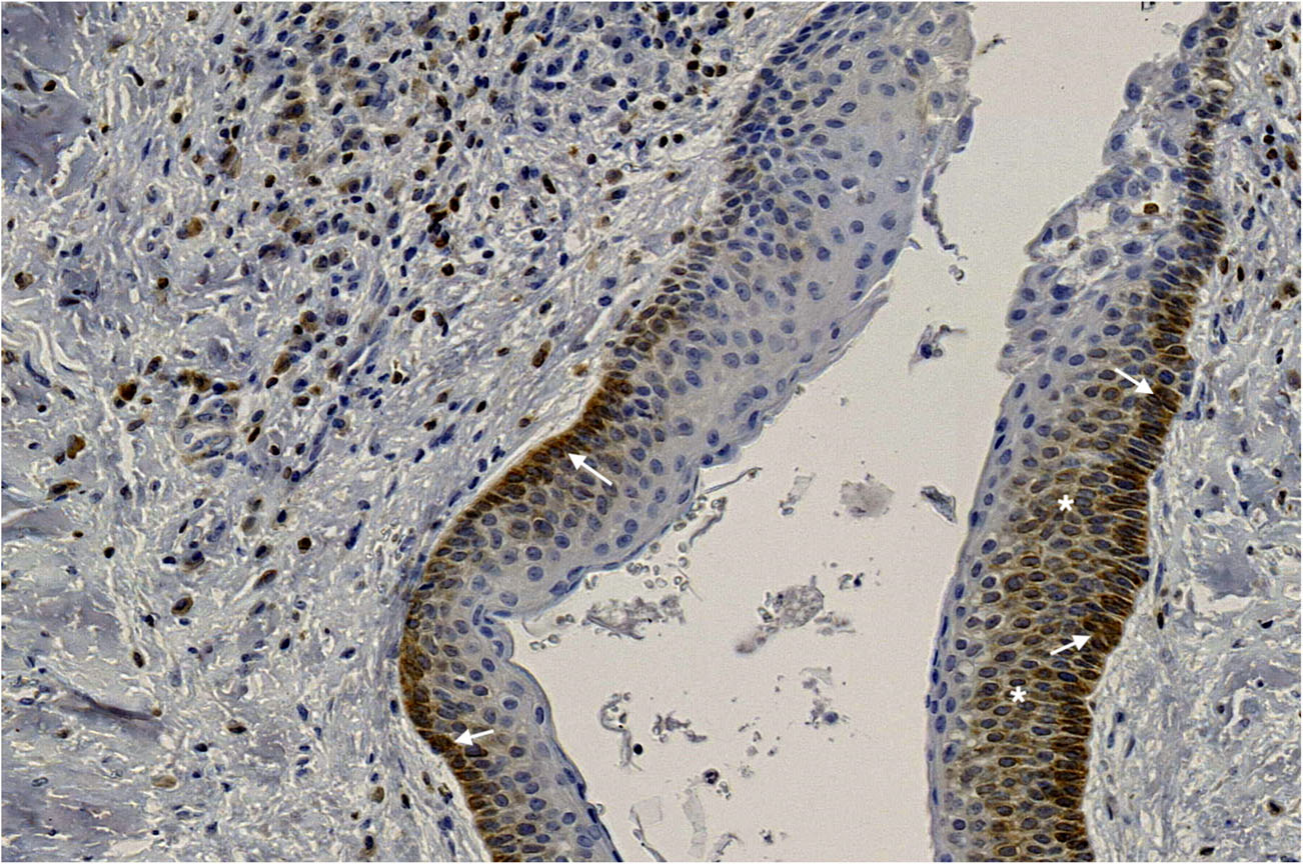
bcl-2—membranous-cytoplasmic brown staining in the basal (arrows) and suprabasal (asterisks) layers of the epithelial lining of OKC [× 60]

**Fig. 3:**
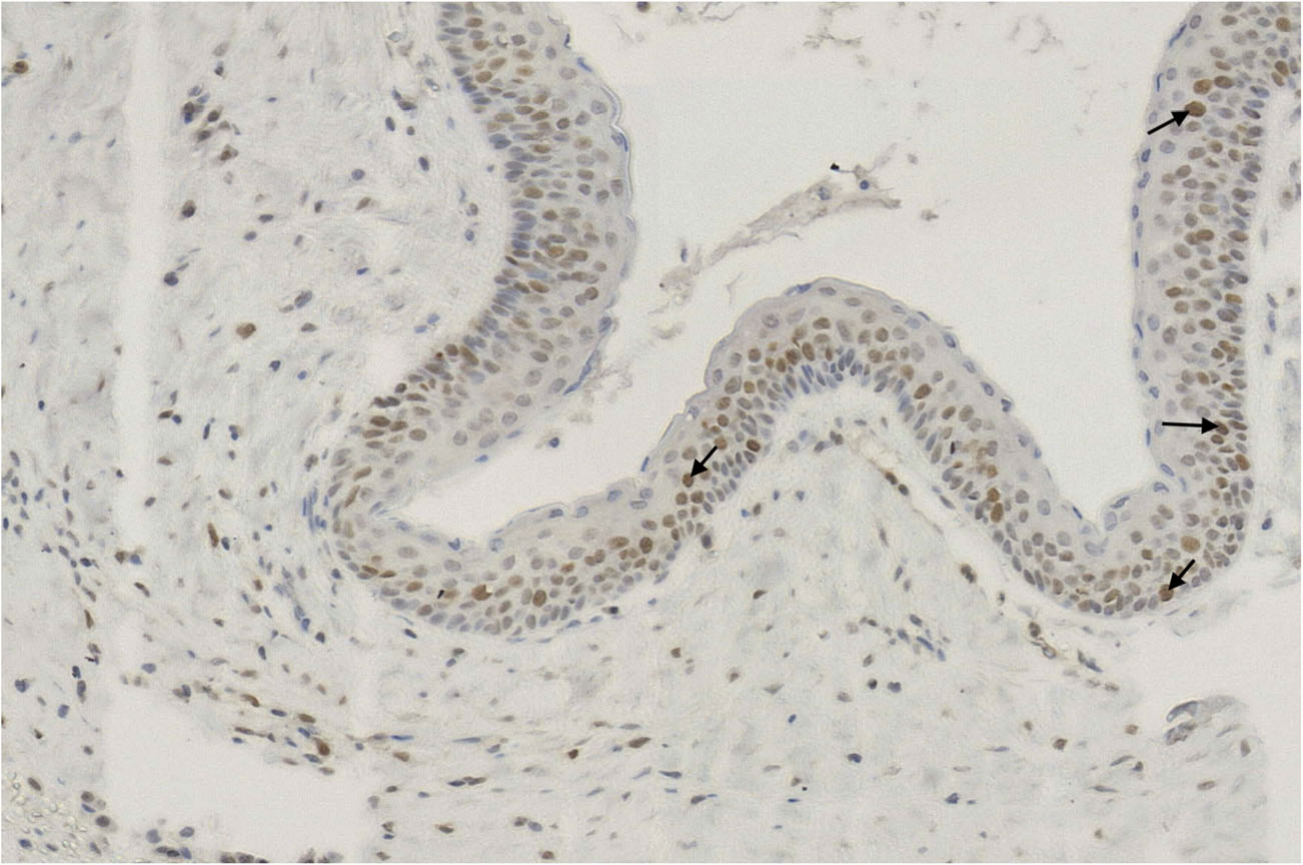
PCNA—nuclear brown staining (arrows) in the epithelial lining of OKC [× 60]

**Fig. 4:**
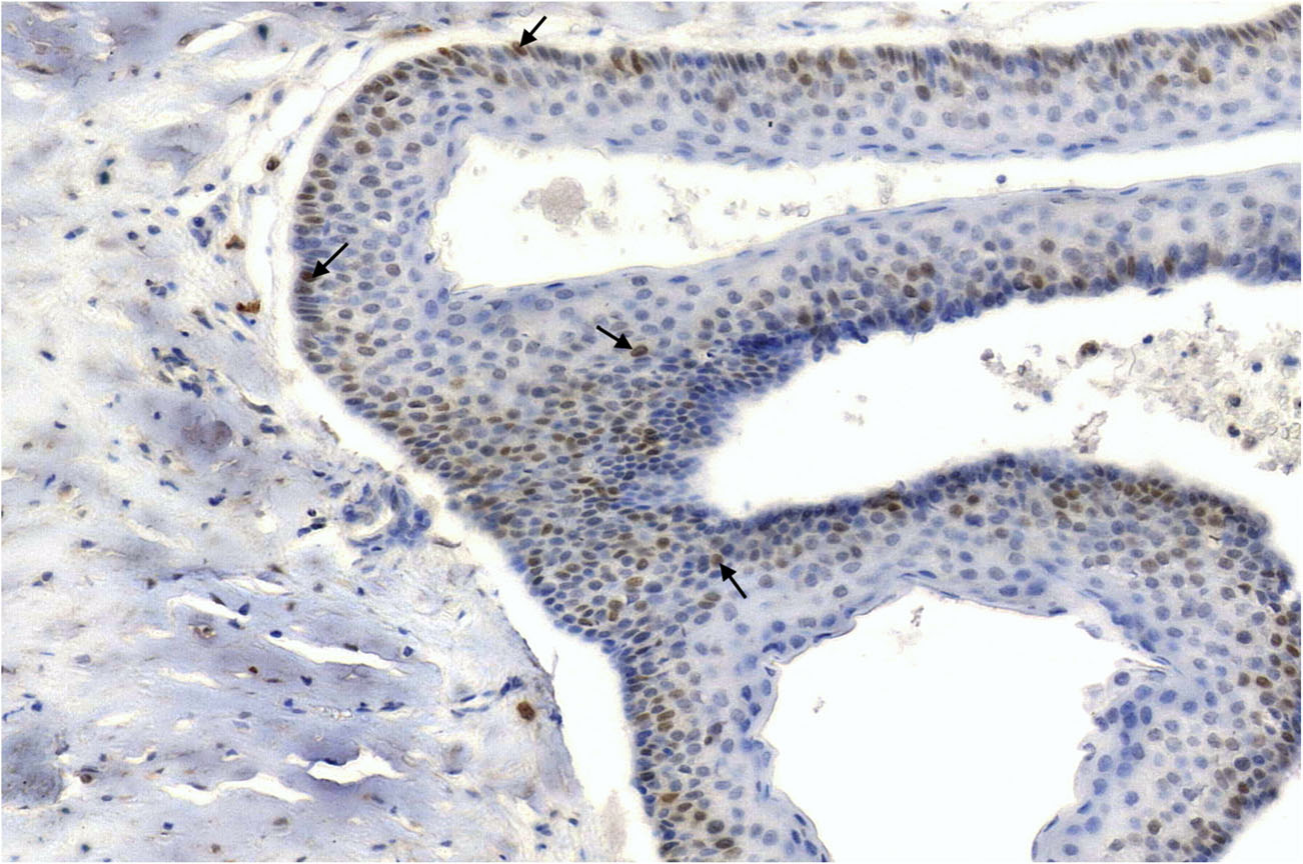
p53—nuclear brown staining (arrows) in the epithelial lining of OKC [× 60]

Details of immunoexpression of individual molecules are given in Figs. 1, 2, 3, and 4. We found no significant differences in the expression of any marker between recurrent and non-recurrent OKCs (Tables 2 and 3). Complete characteristics of the recurrent cases are presented in Table 4.

**Table 2:** Immunohistochemical expression of COX-2 and bcl-2 in the epithelial component of OKCs with hazard risks for recurrence

		Recurrent cysts (*n* = 12)	Non-recurrent cysts (*n* = 29)	*P* value (Fisher’s exact test)	Cox proportional hazard model	
					HR (95% CI)	*P* value
COX-2	0% positive cells	1 (8.3%)	3 (10.34%)	0.776	1.00	
	1–25% positive cells	3 (25.0%)	12 (41.3%)		0.812 (0.084–7.817)	0.857
	26–50% positive cells	4 (33.3%)	8 (27.6%)		1.406 (0.157–12.601)	0.76
	Over 50% positive cells	4 (33.3%)	6 (20.7%)		1.726 (0.193–15.445)	0.626
bcl-2	0% positive cells	1 (8.3%)	10 (34.5%)	0.274	1.00	
	1–25% positive cells	9 (75.0%)	13 (44.9%)		5.396 (0.681–42.765)	0.111
	26–50% positive cells	2 (16.7%)	4 (13.8%)		4.327 (0.392–47.775)	0.232
	Over 50% positive cells	0 (0.0%)	2 (6.9%)		NR	

*NR*, no recurrence; *HR*, hazard ratio; *CI*, confidence interval

**Table 3:** Immunohistochemical expression of PCNA and p53 in the epithelial component of OKCs with hazard risks for recurrence

	Recurrent cysts (*n* = 12)		Non-recurrent cysts (*n* = 29)		*P* value (Mann-Whitney test)	Cox proportional hazard model	
	Median (range) [%]	Mean (SD) [%]	Median (range) [%]	Mean (SD) [%]		HR (95% CI)	*P* value
PCNA	24 (17.62–35)	27.25 (14.35)	19.5 (16–29)	23.07 (9.62)	0.282	1.039 (0.982–1.098)	0.185
p53	12 (9.75–14.25)	13.5 (7.29)	11 (5–16)	11.47 (6.96)	0.414	1.04 (0.958–1.129)	0.346

**Table 4:** Clinicoradiological and histopathological characteristics of the recurrent cases of OKC

No.	Age	Sex	Location	Relapse period (months)	Cyst variant	Size of lesion (mm^2^)	Cortical perforation	Inflammatory score	Number of daughter cysts	Epithelial dysplasia	COX-2 [grade]	bcl-2 [grade]	PCNA [%]	p53 [%]
1	48	F	A-Mn	24	UL	950	(+)	2	0	(−)	2	1	10.5	6
2	36	F	P-Mn	36	UL	760	(−)	0	0	(−)	2	2	13	13
3	15	M	P-Mn	72	ML	1148	(+)	0	0	(−)	3	1	23	11.5
4	19	M	A-Mn	108	ML	950	(+)	1	0	(+)	2	1	24	18
5	36	M	P-Mn	36	ML	594	(+)	0	0	(−)	3	1	13.5	11
6	67	M	P-Mn	60	ML	1064	(+)	0	0	(−)	0	1	19	21
7	29	M	P-Mn	72	UL	700	(−)	2	0	(−)	3	1	24	9
8	58	M	A-Mx	24	UL	375	(+)	3	1	(−)	1	0	38	12.5
9	10	F	A-Mx	24	UL	375	(+)	0	1	(−)	2	1	38	32
10	62	M	A-Mn	24	ML	875	(−)	1	3	(−)	1	1	28	10
11	18	M	A-Mn	18	ML	1350	(+)	0	0	(−)	3	2	62	5.5
12	70	M	A-Mx	84	ML	759	(+)	1	0	(−)	1	1	34	12.5

*A-Mn*, anterior mandible; *P-Mn*, posterior mandible; *A-Mx*, anterior maxilla; *UL*, unilocular; *ML*, multilocular

Spearman correlation analysis showed that expression of COX-2 was negatively correlated with age (*r* = − 0.461; *P* = 0.002) and that expression of PCNA was positively correlated with radiological evidence of cortical perforation (*r* = 0.311; *P* = 0.048). Furthermore, the positive correlation between expression of COX-2 and bcl-2 was revealed (*r* = 0.505; *P* = 0.001). No other significant correlations between expression of individual proteins and clinicoradiological and histopathological features of OKC were discovered.

## Discussion

OKC is a lesion of ambiguous nature. Some OKCs do not recur after marsupialization or other conservative approaches, while some show aggressive behavior and tendency to relapse despite more radical treatment [18]. Several studies have analyzed clinicoradiological features and immunohistochemical expression of various indicators of proliferation and apoptosis in OKC to discover prognostic markers [9, 10, 12, 19], but only a few attempted to employ the time-dependent analysis for determining its potential for recurrence after surgery [8]. The present study retrieved the clinical, radiological, and histopathological data of 41 sporadic cases of OKC and, together with their immunohistochemical expression of COX-2, bcl-2, PCNA, and p53, endeavored to correlate them with the potential for relapse.

Among radiological findings investigated in the current analysis, multilocularity and cyst size were significantly related to recurrence. These results are in line with observations by other authors [9, 19, 20]. It has been demonstrated that smaller OKCs are usually unilocular, whereas larger lesions tend to be multilocular [21, 22]. Most probably, this is the reason why larger and multilocular OKCs are more prone to recur than smaller and unilocular lesions which are easier to enucleate in one piece. Many authors pointed out that recurrence of OKC may be related to inadequate treatment as multilocular and large lesions are difficult to access and the fragments are easily overlooked in the course of surgical procedure [19, 20]. However, in some reports, size and multilocularity were not associated with recurrence of OKC [8, 23].

To the best of our knowledge, in none of the previous reports, radiological presence of cortical perforation was examined as a potential prognostic factor in OKC. This imaging feature is occasionally observed in OKC [1] and may be a manifestation of destructive clinical behavior of a lesion. Hence, it is more often discovered in aggressive odontogenic tumors like ameloblastoma than in benign odontogenic cysts. Although neoplastic nature of OKC is currently not supported [2, 3], a few studies demonstrated its potential to infiltrate through the medullary spaces and erode cortical bone which is similar to ameloblastoma [24, 25]. Cortical perforation may indicate the involvement of adjacent soft tissues which, in turn, may lead to incomplete removal of the cyst and subsequent relapse. In the current study, cortical perforation has been identified as a very significant prognostic factor as its presence in radiologic examination is associated with 7.8 times higher risk of recurrence. Hence, conservative treatment (which was implemented in our study) may be insufficient to remove the entire lining of OKC and the remaining tissue may lead to recurrence. Consequently, some authors postulated that to prevent relapse, the overlying attached mucosa may need to be excised in conjunction with enucleation or even partial bone resection could be applied in some more extensive cases [7].

In the current study, the site of involvement was not related to recurrence and this finding is consistent with that of Yagyuu et al. [19] and with that of Naruse et al. [26]. Likewise, results of thorough analysis of 6427 OKC cases performed by Chrcanovic and Gomez [20] indicated that there are no apparent differences in the recurrence rates between lesions located in the maxilla and in the mandible. Interestingly, Myoung et al. [27] found no significant difference between recurrence rates of OKC occurring in the maxilla and occurring in the mandible, but in further detailed investigation of recurrence rates of six subdivided jaw regions, they revealed that mandibular molar region was markedly more prone to relapse. They suggested that the differences in recurrence rate might be related to the area’s surgical accessibility [27]. Indeed, the posterior mandibular region as well as mandible ramus has been recognized to present the difficulty of complete cyst removal [28].

OKC may show small daughter (satellite) cysts [2], and according to Pavelić et al. [29], these are present in 20.5% cases of OKC. Correspondingly, in the current series, daughter cysts were found in 24.4% of cases. Their occurrence is more common in NBCCS-associated cases of OKC [2]. Some authors demonstrated that the presence of daughter cysts may be related to the recurrence of OKC [27]; nonetheless, we did not find such a correlation. Similar to our results, the presence of daughter cysts was also not associated with recurrence rates of OKC in the series investigated by Kuroyanagi et al. [8] and Naruse et al. [26]. Previous studies reported that the presence of daughter cysts is significantly associated with a high frequency of allelic loss in tumor suppressor genes, thus suggesting its neoplastic nature [16]. Currently, however, experts do not support the neoplastic background of OKC development [2, 3].

Many attempts have been made to elucidate biological behavior of OKC, and investigations have tried to identify various molecular markers as potential factors indicating propensity of OKC towards relapse. It has been suggested that COX-2 may contribute to the biological profile of OKC, playing a role in the biological regulation of its epithelial lining [30]. Wang et al. [13] demonstrated that COX-2 is at least partially involved in the mechanism of progression of OKC and that its immunoexpression significantly decreases after decompression, one of the surgical options of treatment that results in shrinking of the cyst size. COX-2 levels have been found to be elevated in majority of tumors, and its prognostic significance has been demonstrated in several of them, including head and neck cancer [31]. Although in the current study immunopositivity of COX-2 has been demonstrated in majority of cases, we did not find that the level of its expression has any prognostic significance in OKC. Other studies elucidating association between COX-2 expression and recurrence in OKC are scarce but produced the same conclusions [12]. Mendes et al. [12] suggested that lack of association between the expression of COX-2 and clinical features shows that the pathogenic mechanism involved in OKC is not “clinical-related” and it unveils an intrinsic characteristic of OKC that bears no association either with its syndromic or primary nature, thus substantiating previous molecular studies establishing the neoplastic nature of this lesion [12, 32–34].

COX-2 is known to increase the level of bcl-2 which, in turn, suppresses apoptosis by preventing activation of caspases responsible for cell death [11, 35]. Studies on bcl-2 expression in OKC are few in number and most of them are confined to primary lesions. Vered et al. [36] demonstrated that bcl-2 expression in primary sporadic and syndromic OKCs was similar to solid ameloblastoma but significantly higher in comparison with clinically benign radicular, dentigerous, and orthokeratinized odontogenic cysts. These observations are consistent with other authors [14] and may suggest that biological behavior of OKC is different from other common jaw cysts. It has also been demonstrated that bcl-2 immunopositvity is significantly higher in syndromic OKCs than in their sporadic counterparts [37]. In the current study, we found positive correlation between bcl-2 and COX-2 which corroborates the conclusions of other authors [11, 35]. We did not, however, reveal significant association between bcl-2 immunopositivity and recurrence of OKC.

PCNA has been shown to be a useful marker for the proliferating fraction of cells in various head and neck tumors [38]. By employing PCNA staining, epithelial lining of OKC has been demonstrated to have a comparable proliferative potential to that of ameloblastoma [39]. High expression of PCNA in OKC may reflect the high level of cell proliferative activity in the lining epithelium which supported the outdated concept of the neoplastic nature of this lesion [9, 40]. In the current study, it has been demonstrated that there is a positive correlation between expression of PCNA and presence of cortical perforation which may serve as a strong prognostic factor for OKC recurrence. However, we have found no significant association between PCNA immunopositivity and recurrence of OKC. To the best of our knowledge, there are no other studies available which assessed PCNA as a potential marker of OKC relapse, with the exception of el Murtadi et al. [10] who discovered that mean PCNA count in recurrent OKCs was significantly lower in comparison with that in *de novo* cases. They suggested that this trend indicates that the stimulus for proliferation is maximum around the time of occurrence of the primary cyst and does not appear to be continuous throughout life as it decreases in intensity with time [10]. It is difficult to compare the results of the study by el Murtadi et al. [10] with ours, as we did not analyze primary OKCs vs. recurrences of OKCs but all our cases were primary which were subdivided into recurrent and non-recurrent cases. Moreover, in contrast to the current study, el Murtadi et al. [10] also included syndromic cases to their analysis.

Results of some investigations showed that immunoexpression of p53 strongly correlates with the recurrence of various head and neck tumors [41]; however, the prognostic value of p53 immunostaining in oral cancer has been disputed [42]. With regard to OKC, only Razavi et al. [43] demonstrated that evaluation of p53 expression at the time of diagnosis may be useful for the prediction of recurrence. Other authors, including Lombardi et al. [44], Gurgel et al. [45], and Kuroyanagi et al. [8], did not find statistically significant differences in expression of p53 between recurrent and non-recurrent OKCs, and their results are consistent with ours. These discrepancies may result from the fact that OKC can show different clinical behaviors, which was also pointed out by Razavi et al. [43].

Irrespective of the current non-neoplastic concept of OKC pathobiology [2], from the clinical point of view, some OKC cases show an indolent course typical for most of odontogenic cysts, while others demonstrate aggressive behavior similar to that of ameloblastoma. Thus, further research on larger number of cases which may allow to stratify these clinically distinct OKC cases is essential. In this material encompassing a relatively small number of cases, assessment of immunoexpression of COX-2, bcl-2, PCNA, and p53 was not useful to identify cyst with more aggressive phenotype and higher tendency to relapse.

It can be concluded that some radiological features of sporadic OKCs, such as larger size, multilocularity, and evidence of cortical perforation, may indicate higher risk of recurrence. However, the significance of the association between these radiographic characteristics of OKC and its tendency to relapse requires confirmation in large-scale studies. We also found that expression of PCNA significantly correlates with the radiological presence of cortical perforation, and that there is a significant positive correlation between immunopositivity for COX-2 and bcl-2 and significant negative correlation between immunopositivity for COX-2 and age. Nevertheless, further investigation is still necessary to reveal the precise molecular factors behind OKC recurrence.

## References

[1] Philipsen HP (2005) Keratocystic odontogenic tumour. In: Barnes L, Evenson JW, Reichart P, Sidransky D (eds) Pathology and genetics of head and neck tumours. IARC Press, Lyon, pp 306–307

[2] Speight P, Devilliers P, Li TJ, Odell EW, Wright JM (2017) Odontogenic keratocyst. In: El-Naggar AK, Chan JKC, Grandis JR, Takata T, Slootweg PJ (eds) WHO classification of head and neck tumours. IARC Press, Lyon, pp 235–236

[3] Wright JM, Vered M (2017). Update from the 4th edition of the World Health Organization classification of head and neck tumours: odontogenic and maxillofacial bone tumours. Head Neck Pathol.

[4] Pitak-Arnnop P (2010). Enucleation of keratocystic odontogenic tumours: study interpretation, technical refinement and future research. Clin Oral Invest.

[5] Antonoglou GN, Sȧndor GK, Koidou VP, Papageorgiou SN (2014). Non-syndromic and syndromic keratocystic odontogenic tumours: systematic review and meta-analysis of recurrences. J Craniomaxillofac Surg.

[6] Kaczmarzyk T, Mojsa I, Stypulkowska J (2012). A systematic review of the recurrence rate for keratocystic odontogenic tumour in relation to treatment modalities. Int J Oral Maxillofac Surg.

[7] Al-Moraissi EA, Dahan AA, Alwadeai MS, Oginni FO, Al-Jamali JM, Alkhutari AS, Al-Tairi NH, Almaweri AA, Al-Sanabani JS (2017). What surgical treatment has the lowest recurrence rate following the management of keratocystic odontogenic tumor?: a large systematic review and meta-analysis. J Craniomaxillofac Surg.

[8] Kuroyanagi N, Sakuma H, Miyabe S, Machida J, Kaetsu A, Yokoi M, Maeda H, Warnakulasuriya S, Nagao T, Shimozato K (2009). Prognostic factors for keratocystic odontogenic tumor (odontogenic keratocyst): analysis of clinico-pathologic and immunohistochemical findings in cysts treated by enucleation. J Oral Pathol Med.

[9] Ibrahim N, Nazimi AJ, Ajura AJ, Nordin R, Latiff ZA, Ramli R (2016). The clinical features and expression of bcl-2, cyclin D1, p53, and proliferating cell nuclear antigen in syndromic and nonsyndromic keratocystic odontogenic tumor. J Craniofac Surg.

[10] el Murtadi A, Grehan D, Toner M, McCartan BE (1996). Proliferating cell nuclear antigen staining in syndrome and nonsyndrome odontogenic keratocyst. Oral Surg Oral Med Oral Pathol Oral Radiol Endod.

[11] Mendes RA, Carvalho JFC, van der Waal I (2009). An overview on the expression of cyclooxygenase-2 in tumors of the head and neck. Oral Oncol.

[12] Mendes RA, Carvalho JFC, van der Waal I (2011). Potential relevance of cyclooxygenase-2 expression in keratocystic odontogenic tumours—an immunohistochemical study. J Oral Pathol Med.

[13] Wang J, Zhang X, Ding X, Xing S, Li H, Zang W, Wang L, Wu H (2013). Cyclooxygenase-2 expression in keratocystic odontogenic tumour decreased following decompression. Mol Clin Oncol.

[14] Piattelli A, Fioroni M, Rubini C (1998). Differentiation of odontogenic keratocysts from other odontogenic cysts by the expression of bcl-2 immunoreactivity. Oral Oncol.

[15] Ogden GR, Chisholm DM, Kiddie RA, Lane DP (1992). p53 protein in odontogenic cyst: increased expression in some odontogenic keratocysts. J Clin Pathol.

[16] Agaram NP, Collins BM, Barnes L, Lomago D, Aldeeb D, Swalsky P, Finkelstein S, Hunt JL (2004). Molecular analysis to demonstrate that odontogenic keratocysts are neoplastic. Arch Pathol Lab Med.

[17] Medes RA, Carvalho JFC, van der Waal I (2010). Characterization and management of the keratocystic odontogenic tumor in relation to its histopathological and biological features. Oral Oncol.

[18] Leonardi R, Perrotta RE, Crimi S, Matthews JB, Barbato E, dos Santos JN, Rusu M, Bufo P, Bucci P, Pannone G (2015). Differential expression of TLR3 and TLR4 in keratocystic odontogenic tumor (KCOT): a comparative immunohistochemical study in primary, recurrent, and nevoid basal cell carcinoma syndrome (NBCCS)-associated lesions. J Craniomaxillofacial Surg.

[19] Yagyuu T, Kirita T, Sasahira T, Moriwaka Y, Yamamoto K, Kuniyasu H (2008). Recurrence of keratocystic odontogenic tumor: clinicopathological features and immunohistochemical study of the Hedgehog signaling pathway. Pathobiology.

[20] Chrcanovic BR, Gomez RS (2017). Recurrence probability for keratocystic odontogenic tumors: an analysis of 6427 cases. J Craniomaxillofac Surg.

[21] Avril L, Lombardi T, Ailianou A, Burkhardt K, Varoquaux A, Scolozzi P, Becker M (2014). Radiolucent lesions of the mandible: a pattern-based approach to diagnosis. Insights Imaging.

[22] Finkelstein MW, Hellstein JW, Lake KS, Vincent SD (2013). Keratocystic odontogenic tumor: a retrospective analysis of genetic, immunohistochemical and therapeutic features. Proposal of a multicenter clinical survey tool. Oral Surg Oral Med Oral Pathol Oral Radiol.

[23] Forssel K, Forssel H, Kahnberg KE (1988). Recurrence of keratocysts. A long-term follow-up study. Int J Oral Maxillofac Surg.

[24] Tekkesin MS, Mutlu S, Olgac V (2011). The role of RANK/RANKL/OPG signalling pathways in osteoclastogenesis in odontogenic keratocysts, radicular cysts, and ameloblastomas. Head Neck Pathol.

[25] de Matos FR, de Moraes M, das Neves Silva EB, Galvão HC, de Almeida Freitas R (2013). Immunohistochemical detection of receptor activator nuclear κB ligand and osteoprotegerin in odontogenic cysts and tumors. J Oral Maxillofac Surg.

[26] Naruse T, Yamashita K, Yanamoto S, Rokutanda S, Matsushita Y, Sakamoto Y, Sakamoto H, Ikeda H, Ikeda T, Asahina I, Umeda M (2017). Histopathological and immunohistochemical study in keratocystic odontogenic tumors: predictive factors of recurrence. Oncol Lett.

[27] Myoung H, Hong SP, Hong SD, Lee JI, Lim CY, Choung PH, Lee JH, Choi JY, Seo BM, Kim MJ (2001). Odontogenic keratocyst: review of 256 cases for recurrence and clinicopathologic parameters. Oral Surg Oral Med Oral Pathol Oral Radiol Endod.

[28] Partridge M, Towers JF (1987). The primordial cyst (odontogenic keratocyst): its tumour-like characteristics and behaviour. Br J Oral Maxillofac Surg.

[29] Pavelić B, Katunarić M, Segović S, Karadole MC, Katanec D, Saban A, Puhar I (2014). The incidence of satellite cysts in keratocystic odontogenic tumors. Coll Antropol.

[30] Mendes RA, Carvalho JFC, van der Waal I (2011). A comparative immunohistochemical analysis of COX-2, p53, and Ki-67 expression in keratocystic odontogenic tumors. Oral Surg Oral Med Oral Pathol Oral Radiol Endod.

[31] Yang B, Jia L, Guo Q, Ren H, Hu Y, Xie T (2016) Clinicopathological and prognostic significance of cyclooxygenase-2 expression in head and neck cancer: a meta-analysis*.* Oncotarget 7:47265–47277 10.18632/oncotarget.10059 PMC5216940 27323811

[32] Shear M (2002). The aggressive nature of the odontogenic keratocyst: is it a benign cystic neoplasm? Part 2: proliferation and genetic studies. Oral Oncol.

[33] Katase N, Nagatsuka H, Tsujigiwa H, Gunduz M, Tamamura R, Pwint HP, Rivera RS, Nakajima M, Naomoto Y, Nagai N (2007). Analysis of the neoplastic nature and biological potential of sporadic and nevoid basal cell carcinoma syndrome-associated keratocystic odontogenic tumor. J Oral Pathol Med.

[34] Lo Muzio L, Staibano S, Pannone G, Bucci P, Nocini PF, Bucci E, De Rosa G (1999). Expression of cell cycle and apoptosis-related proteins in sporadic odontogenic keratocysts and odontogenic keratocysts associated with the nevoid basal cell carcinoma syndrome. J Dent Res.

[35] Mohan S, Epstein JB (2003). Carcinogenesis and cyclooxygenase: the potential role of COX-2 inhibition in upper aerodigestive tract cancer. Oral Oncol.

[36] Vered M, Peleg O, Taicher S, Buchner A (2009). The immunoprofile of odontogenic keratocyst (keratocystic odontogenic tumor) that includes expression of PTCH, SMO, GLI-1 and bcl-2 is similar to ameloblastoma but different from odontogenic cysts. J Oral Pathol Med.

[37] Kolář Z, Geierová M, Bouchal J, Pazdera J, Zbořil V, Tvrdý P (2006). Immunohistochemical analysis of the biological potential of odontogenic keratocysts. J Oral Pathol Med.

[38] Pich A, Chiusa L, Navone R (2004). Prognostic relevance of cell proliferation in head and neck tumors. Ann Oncol.

[39] Shahela T, Aesha S, Ranganathan K, T R, Roa KUD, Joshua E, Ahmed AS, Chittamsetty H (2013) Immunohistochemical expression of PCNA in epithelial linings of selected odontogenic lesions. J Clin Diagn Res 7:2615–2618 10.7860/JCDR/2013/5824.3629 PMC3879850 24392421

[40] Seyedmajidi M, Nafarzadeh S, Siadati S, Shafaee S, Bijani A, Keshmiri N (2013). p53 and PCNA expression in keratocystic odontogenic tumors compared with selected odontogenic cysts. Int J Mol Cell Med.

[41] Ball VA, Righi PD, Tejada E, Radpour S, Pavelic ZP, Gluckman JL (1997). p53 immunostaining of surgical margins as a predictor of local recurrence in squamous cell carcinoma of the oral cavity and oropharynx. Ear Nose Throat J.

[42] Gröbe A, Hanken H, Al-Dam A, Cachovan G, Smeets R, Krohn A, Clauditz T, Grob T, Simon R, Sauter G, Kluwe L, Heiland M, Blessmann M (2014). P53 immunohistochemical expression does not correlate with clinical features in 207 carcinomas of the oral cavity and in the head and neck region. Clin Oral Invest.

[43] Razavi SM, Chalice S, Torabinia N (2014). Investigation of clinicopathological parameters alongside with p53 expression in primary and recurrent keratocystic odontogenic tumours. Malays J Pathol.

[44] Lombardi T, Odell EW, Morgan PR (1995). p53 immunohistochemistry of odontogenic keratocysts in relation to recurrence, basal-cell budding and basal-cell naevus syndrome. Arch Oral Biol.

[45] Gurgel CA, Ramos EA, Azevedo RA, Sarmento VA, da Silva Carvalho AM, dos Santos JN (2008). Expression of Ki-67, p53 and p63 proteins in keratocystic odontogenic tumours: an immunohistochemical study. J Mol Histol.

